# Oral Lichen Planus in a Pediatric Patient: A Novel Therapeutic Approach

**Published:** 2017-03

**Authors:** Gaurav Sharma, Divesh Sardana, Puneeta Vohra, Shweta Rehani, Archna Nagpal

**Affiliations:** 1 Professor, Department of Oral Medicine and Radiology, S.R. Dental College, Faridabad, Haryana, India; 2 Senior Resident, Division of Pediatric Dentistry, Center for Dental Education and Research (CDER), All India Institute of Medical Sciences (AIIMS), New Delhi, India; 3 Reader, Department of Oral Medicine and Radiology, SGT Dental College, Gurgaon, Haryana, India; 4 Reader, Department of Oral Pathology, S.R. Dental College, Faridabad, Haryana, India; 5 Professor, Department of Oral Medicine and Radiology, P.D.M. Dental College, Bahadurgarh, Haryana, India

**Keywords:** Lichen Planus, Oral, Aloe Vera Gel, Retinoids, Therapeutics

## Abstract

Lichen planus is a mucocutaneous disease, predominantly affecting the middle-aged individuals and may be associated with a plethora of signs and symptoms related to the skin, scalp, nails and mucous membranes. The definitive etiology of lichen planus is not yet known and no therapeutic modality has yet been universally accepted. Lichen planus in pediatric patients is a rare phenomenon and its presence in the oral mucosa is even rarer. The aim of this article is to present a rare case of a symptomatic oral lichen planus (OLP) occurring in a 12-year old child that was managed successfully with a novel sequential modality of topical retinoids followed by aloe vera gel application.

## INTRODUCTION

Lichen planus is a chronic mucocutaneous cell-mediated immune disorder that involves the Langerhans cells, T-lymphocytes and macrophages. Both CD4 helper and CD8 cytotoxic cells have been found in increased numbers in this condition that may contribute to basal epithelial cell layer death [1,2]. Various factors like genetic predisposition, infective agents, systemic diseases, graft versus host disease, drug reactions, hypersensitivity to dental materials and vitamin deficiencies have been implicated in pathogenesis of lichen planus but their role has not been proven conclusively [1]. Cutaneous lichen planus in pediatric patients is a seldom reported condition; whereas, the presence of oral lichen planus (OLP) in pediatric patients is exceedingly rare. The exact incidence of OLP in pediatric patients is unknown, although Alam and Hamburger [3] reported a prevalence of 0.5% for OLP in pediatric patients with pre-existing diagnosed cutaneous lichen planus. Kumar et al. [4] observed only one pediatric OLP case in 25 cutaneous lichen planus patients while Xue et al. [5] documented four cases of OLP in children out of 674 cutaneous lichen planus cases. Woo et al. [6] summarized the criteria for juvenile/pediatric lichen planus to be a biopsy-proven OLP in patients less than 20 years of age with no probability of oral lichenoid reaction. The present article, thus, is a rare case report of symptomatic OLP in a 12-year-old female patient, that was successfully managed with a novel therapeutic sequential modality of application of topical retinoids initially followed by aloe vera gel application. Although there are case reports regarding the management of OLP with aloe vera, the present case is rare in reporting the optimal efficacy of aloe vera in a pediatric patient. Significant clinical resolution and symptomatic relief were observed during the six-month follow-up.

## CASE REPORT

A 12-year-old female patient was referred to the Department of Oral Medicine and Diagnosis with a complaint of burning sensation in the mouth while eating spicy foods started three months earlier. The burning sensation lasted for a few minutes and was relieved on its own.

**Fig. 1:**
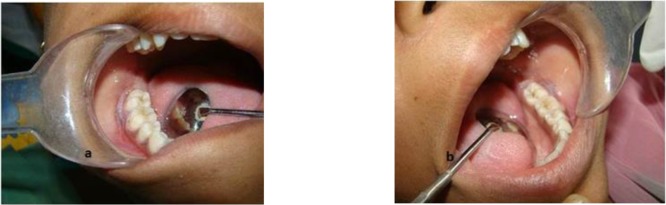
Grayish-white patch with striations seen on (a) right gingiva and vestibule and (b) left gingiva and vestibule

There was no history of recent change in toothpaste, use of mouthwashes or fluoride application. No history of change in food habits in the past was elicited. The patient had not undergone any kind of treatment. The patient did not report any pruritus, skin eruptions, burning micturition, eye lesions or joint pains. No history of any medication usage or previous hospitalization was documented. There was no contributory medical or family history. The patient had not undergone any dental procedure in the past. There was no evidence of lesions on dermatological examination. No obvious nail or scalp changes were evident. Cervical lymph nodes were non-palpable. On intraoral examination, the patient’s oral hygiene was fair. Dental examination showed no decayed or restored teeth. Soft tissue examination revealed presence of irregular 2×2cm grayish white patches with peripherally radiating white striae on the gingiva and vestibular area of the permanent mandibular molars bilaterally (Fig. 1). These patches were non-scrapable, non-tender and non-indurated.

**Fig. 2:**
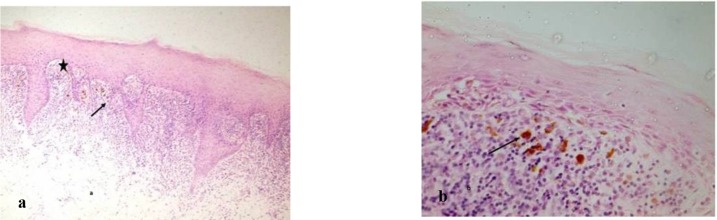
Photomicrograph showing (a) hyperplastic parakeratinized stratified squamous epithelium with saw tooth-shaped rete-ridges and juxta-epithelial band of lymphocytes (H&E stain, x10). The arrow indicates basal cell degeneration; the star indicates Max- Joseph space; (b) atrophic epithelium and melanin incontinence indicated by arrow (H&E stain, x40).

There was no bleeding of lesions on manipulation. The buccal mucosa, tongue, floor of the mouth, hard palate and soft palate were clinically normal. A provisional diagnosis of lichen planus was made and the patient’s parents were made aware of the condition. An incisional biopsy was done after obtaining written informed consent from the patient’s parents. The histopathological features showed hyperplastic parakeratinized stratified squamous epithelium with underlying connective tissue (Fig. 2). The epithelium exhibited acanthosis and basal cell degeneration with few saw tooth-shaped rete-ridges and fewer areas showing disruption in basement membrane and intraepithelial lymphocytes (Fig. 2). The juxta-epithelial connective tissue showed a band of lymphocyte infiltration. The histopathological features confirmed the provisional diagnosis of OLP. No similar mucosal findings were observed in the subsequent intraoral examination of the patient’s parents and her sibling. Routine hematological and liver function tests of the patient were within the normal range.

Oral prophylaxis was done and the patient was placed on chlorohexidine mouth rinse. Initially, 0.2% topical steroid (triamcinolone acetonide) was prescribed for the patient for two weeks along with topical antifungals (1% clotrimazole). However, after two weeks, the patient was still symptomatic with no visible improvement. Thus, all the medications were suspended and 0.05% topical tretinoin was prescribed to be applied twice a day for two weeks. The patient showed a remarkable improvement in symptoms and clinical appearance. However, considering the adverse effects of retinoids in case of long-term use in a pediatric patient, retinoids were suspended and the patient was subsequently advised to use aloe vera gel topically twice a day for one month.

**Fig. 3:**
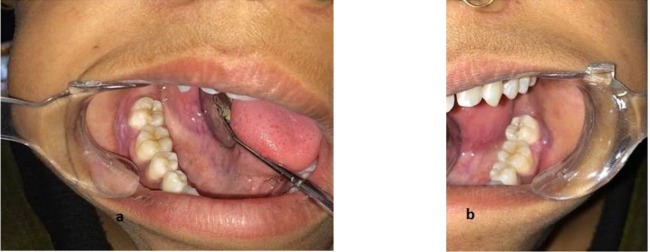
Post-treatment, healed lesions observed in (a) right gingiva and vestibule and (b) left gingiva and vestibule

The patient was asymptomatic and there was clinical resolution of lesions at the one-month visit (Fig. 3). Aloe vera was subsequently discontinued. The patient and her parents were counselled regarding the condition. They were advised and motivated for a mandatory semi-annual check-up irrespective of her asymptomatic state and were made aware of the possible complications.

The patient was asymptomatic at her next semi-annual visit. The prevalence of lichen planus varies from 0.3 to 0.8% and it typically affects the skin, nails, scalp and mucous membranes [7]. The occurrence of cutaneous lichen planus in children is a rare phenomenon [2,4,8,9]. OLP in pediatric patients is, however, reported rarely as compared to cutaneous lichen planus [7]. The paucity of pediatric OLP case reports may be attributed to lack of clinical recognition by general practitioners, low incidence of autoimmune diseases in children, poor oral hygiene camouflaging the lesion and lack of awareness of patient and patient’s parents [6]. A slight ethnic predilection in the tropics and a female predilection for OLP in pediatric patients were reported by Woo et al [6]; whereas, Chatterjee et al. [10] did not observe any gender predilection in their study.

OLP shows variability in clinical presentation ranging from classical white, bilateral, symmetrical reticular pattern to erythematous and painful erosive patches to melanotic pigmented areas. There is, however, conflicting data on the similarity of clinical presentation of OLP in pediatric patients with adults. De Moraes et al. [7] advocated atypical presentation of OLP in pediatric patients as compared to adults; whereas, Chatterjee et al. [10] found classical presentation of OLP in their study of 22 cases of OLP in children. Similar to adults, buccal mucosa is the most common intraoral site of involvement by OLP in pediatric patients followed by the tongue [10]. In an extensive review, Woo et al. [6] concluded that asymptomatic reticular variety is the predominant form of OLP in pediatric patients. In the present case, the patient presented clinically with bilateral white patches. The list of differential diagnoses included discoid lupus erythematosus, morsicatio buccarum, auto-immune bullous diseases, and allergic gingivostomatitis. Considering the good general health of our patient, absence of systemic findings and a classical appearance of bilateral grayish-white non-scrapable lesions with peripherally radiating white striae (resembling Wickham striae), a provisional diagnosis of OLP was advocated, which was further confirmed by histopathological examination.

Lichenoid reaction was ruled out as there was no history of medication or any dental restoration. The possibility of familial lichen planus, although extremely rare, was excluded as the patient’s parents were examined and had no evidence of OLP. De Moraes et al. [7] reported a possible occurrence of OLP with complications associated with hepatitis B vaccination. However, our patient had received hepatitis B vaccination at the age of one year, thus, eliminating the possibility of complication. Moreover, the patient’s liver function tests were normal, thus, ruling out the possibility of hepatitis B and hepatitis C infection. A majority of pediatric patients with OLP have concomitant cutaneous lichen planus. However, in the present case, no cutaneous lesions were observed.

Various topical and systemic therapeutic modalities have been proposed for OLP. The mainstay of management of OLP currently remains topical corticosteroids. Other topical treatment modalities suggested include retinoids, cyclosporines and tacrolimus. Systemic medications such as corticosteroids, retinoids, griseofulvin, hydroxychloroquine, azathioprine, mycophenolate mofetil and dapsone have also been proposed. Miscellaneous therapeutic modalities like psoralen with long-wave ultraviolet light, reflexotherapy, surgery and interferons have also been associated with varying results [11].

Unlike our symptomatic case, most of the documented cases of pediatric OLP have been asymptomatic and detected incidentally on a routine intra-oral examination [6]. The management of OLP in a pediatric patient is relatively difficult as compared to adults as the patient’s compliance with regular application of topical medications is challenging. Moreover, once the condition becomes asymptomatic, there is always a likelihood of pediatric patient to discontinue medication. The patient was initially given 1% triamcinolone acetonide topically for two weeks. However, the response to topical steroids in the present case was extremely slow that prompted us for a secondary therapeutic modality.

Topical cyclosporine has often been associated with transient burning sensation and would be expensive on a long-term basis since the patient was from a low socioeconomic status [11]. Tacrolimus is a potent immunosuppressive agent and its topical form has been known to be more permeative in the mucosa as compared to cyclosporine. However, local irritation at the point of application and known history of flare-ups on discontinuation prevented us from prescribing tacrolimus. Painful intralesional steroid injections may not be well tolerated by pediatric patients [11]. Psoralen with long-wave ultraviolet light has oncogenic potential and has known side effects such as nausea, dizziness, eye symptoms and headache [11]. The possibility of giving systemic medications like retinoids, steroids, azathioprine, phenytoin and dapsone was not considered due to young age of the patient.

Topical retinoids are inexpensive, easily and commercially available and safe and provide early symptomatic relief [11]. The patient was, thus, advised topical retinoids and was asked to report immediately if she developed irritation at the point of application, a known side effect of retinoids. The patient responded favorably to therapy. However, topical retinoids are given for a short duration only and there was also a concern regarding the patient’s compliance to topical application as there have been reported cases of recurrence of OLP on discontinuation of retinoids [11]. Aloe vera, an herbal product derived from aloe barbadenses, contains 75 potentially active substances like vitamins, minerals, sugars, amino acids, lignins and enzymes [12]. Aloe vera also possesses good wound healing properties, anti-inflammatory activity, moisturizing action and anti-bacterial activity [13]. Aloe vera has been recently considered in a study as a therapeutic modality for OLP in adults to be more effective than triamcinolone acetonide after eight weeks of follow-up in OLP [14]. Another study showed improved quality of life in patients with OLP with aloe vera application at the 12-week follow-up [15]. Recently, Patil et al. [13] reported successful management of OLP using aloe vera juice and gel application. Local application of aloe vera inhibits the inflammatory process by interfering with the arachidonic acid pathway via cyclooxygenase and also promotes healing [15].

However, there are no case reports or studies depicting the efficacy of aloe vera in OLP in pediatric patients and currently, there is insufficient data on the swift action of aloe vera in OLP. A recent case report on aloe vera’s topical usage showed resolution of clinical symptoms after a period of one month [15]. Thus the authors initially did not consider aloe vera as the primary therapeutic modality since the patient was symptomatic on the first visit. Therefore, the authors used a sequential therapeutic modality that initially provided immediate symptomatic relief (retinoids) and a later safe, easily available, patient compliant, sustainable therapeutic approach (aloe vera). The patient responded favorably to this approach and she was asymptomatic for more than six months.

OLP in pediatric patients should be made asymptomatic swiftly to minimize the possibility of noncompliance to medications. The authors opine that until the prompt and effective action of aloe vera is proven, a sequential therapeutic modality would be a better alternative especially in pediatric patients. Further studies need to be done to detect the efficacy of topical retinoids and aloe vera either alone or in a sequential strategy to manage OLP in pediatric patients. To the best of our knowledge, there are no documented cases of management of OLP in children with this novel combination therapy of topical retinoids initially and maintenance with aloe vera.

OLP has a long-term malignancy potential of 0.9% and its occurrence in pediatric cases implies the possibility of longer existence of lesions in these patients [16]. The probable reason can be a high renewal rate of oral epithelial cells in children. Accordingly, there is a greater need to periodically assess these patients and a diligent management with a constant follow-up should be conducted meticulously. The malignant transformation of OLP, especially in pediatric patients, is still a contentious subject and scientific data are still sparse. Awareness about OLP should be made to children and their parents and a need for a semi-annual examination must be emphasized for such patients.

Pediatric cutaneous lichen planus cases should be referred for mandatory oral medicine consultation by the dermatologists regarding the possibility of OLP. An increased referral is conceivable only with a better coordination between dermatologists and dental practitioners. A lichen planus patient, irrespective of symptoms and clinical presentation, should be monitored by semi-annual check-ups. As OLP is more recalcitrant than cutaneous lichen planus, an early diagnosis can help in early intervention and hence better management. A red and white lesion or reticular appearance in a pediatric patient, especially of Asian descent, should alert the general dental practitioners about the possibility of OLP.

## DISCUSSION

OLP in pediatric patients is an extremely rare entity that can affect the quality of life and can have a long-term psychological impact on patients. The presence of these lesions in a child should alert the dental practitioner for a greater possibility of a malignant transformation. The patients should be motivated and counselled for mandatory semi-annual examination. The authors successfully managed an unusual case of OLP in a child with a novel sequential therapeutic modality of initial topical retinoids and maintenance with aloe vera.
